# Deep Learning Models for Anatomical Location Classification in Esophagogastroduodenoscopy Images and Videos: A Quantitative Evaluation with Clinical Data

**DOI:** 10.3390/diagnostics14212360

**Published:** 2024-10-23

**Authors:** Seong Min Kang, Gi Pyo Lee, Young Jae Kim, Kyoung Oh Kim, Kwang Gi Kim

**Affiliations:** 1 Medical Device R&D Center, Gachon University Gil Hospital, Incheon 21565, Republic of Korea; sm961121@gmail.com; 2 Department of Biomedical Engineering, Gachon University, Seongnam-si 13120, Republic of Korea; pigyo123@gachon.ac.kr; 3 Gachon Biomedical & Convergence Institute, Gachon University Gil Medical Center, Incheon 21565, Republic of Korea; kimyj@gilhospital.com; 4 Department of Internal Medicine, Gachon University Gil Hospital, Incheon 21565, Republic of Korea; kkoimge@gilhospital.com; 5 Department of Biomedical Engineering, Gil Medical Center, Gachon University College of Medicine, Incheon 21565, Republic of Korea

**Keywords:** location inference, deep learning, classification, esophagogastroduodenoscopy, minimum shooting points

## Abstract

Background/Objectives: During gastroscopy, accurately identifying the anatomical locations of the gastrointestinal tract is crucial for developing diagnostic aids, such as lesion localization and blind spot alerts. Methods: This study utilized a dataset of 31,403 still images from 1000 patients with normal findings to annotate the anatomical locations within the images and develop a classification model. The model was then applied to videos of 20 esophagogastroduodenoscopy procedures, where it was validated for real-time location prediction. To address instability of predictions caused by independent frame-by-frame assessment, we implemented a hard-voting-based post-processing algorithm that aggregates results from seven consecutive frames, improving the overall accuracy. Results: Among the tested models, InceptionV3 demonstrated superior performance for still images, achieving an F1 score of 79.79%, precision of 80.57%, and recall of 80.08%. For video data, the InceptionResNetV2 model performed best, achieving an F1 score of 61.37%, precision of 73.08%, and recall of 57.21%. These results indicate that the deep learning models not only achieved high accuracy in position recognition for still images but also performed well on video data. Additionally, the post-processing algorithm effectively stabilized the predictions, highlighting its potential for real-time endoscopic applications. Conclusions: This study demonstrates the feasibility of predicting the gastrointestinal tract locations during gastroscopy and suggests a promising path for the development of advanced diagnostic aids to assist clinicians. Furthermore, the location information generated by this model can be leveraged in future technologies, such as automated report generation and supporting follow-up examinations for patients.

## 1. Introduction

Among gastric cancer screening modalities, esophagogastroduodenoscopy (EGD) is an effective test for improving the detection rate of early gastric cancer [1,2,3]. EGD involves inserting a camera into the upper gastrointestinal tract to directly view the inside of the stomach. However, during an EGD, blind spots, caused by a lack of experience, or errors can reduce its reliability [4,5,6]. The work time of an EGD and the lesion detection rate are proportional, meaning that thoroughly examining all areas of the upper gastrointestinal tract to reduce blind spots can improve EGD reliability [7,8].

Countries worldwide are updating and recommending guidelines for managing gastric cancer, which include minimum imaging points to avoid blind spots. K. Yao et al. proposed a systematic screening protocol to reduce EGD blind spots by selecting 22 minimal imaging points in the upper gastrointestinal tract [9]. According to Bisschops et al., guidelines from the European Society of Gastrointestinal Endoscopy recommend imaging eight endoscopic sites in the upper gastrointestinal tract during an EGD [10].

Increased reliance on medical science has led to greater physician workload and fatigue, higher rates of medical errors, and increased vulnerability to unskilled and inexperienced workers [5,11]. To address these issues, research is continuously being conducted using medical artificial intelligence (AI), with recent studies focusing on deep learning approaches using EGD images and videos. Most AI technologies developed for the EGD field focus on automatically detecting abnormal lesions in the stomach, such as cancer and ulcers. These studies primarily aim to assist doctors in diagnosing diseases by segmenting and highlighting areas of abnormal lesions within the images [12,13,14,15]. The development of a location recognition model is essential for several reasons. While most AI technologies focus on automatically detecting lesions and highlighting these areas, it is equally important in endoscopy to identify the exact location of the lesions within the gastrointestinal tract. A location recognition model provides critical information on where a lesion is situated, which aids in creating a targeted treatment plan. Moreover, such a model enables real-time tracking of the endoscope’s progress, even in the absence of visible lesions, helping to reduce blind spots and ensuring comprehensive examination coverage.

Hirotoshi Takiyama et al. performed image classification of six anatomical locations using a GoogLeNet-based architecture, achieving an accuracy of 97%. However, the study excluded images where the position of the upper gastrointestinal tract was difficult to define and only used still images [16]. Qi He et al. classified images of 12 anatomical locations using the InceptionV3 model, which achieved an accuracy of 82.56%. Unlike previous studies, the study introduced an ‘unqualified’ class to handle images where the anatomical location was difficult to define [17]. Mingjian Sun et al. developed a model for predicting 12 anatomical locations in the gastrointestinal tract using a channel separation-based network. Their model achieved an accuracy of 98.84%, precision of 92.86%, and an F1 score of 92.43% [18]. However, one limitation was the difficulty in distinguishing overlapping features in images containing multiple regions, which made real-time application challenging. This issue was even more pronounced when applied to video data. Additionally, the model’s testing speed was approximately 4 seconds slower than that of the ResNet50 model, making it unsuitable for real-time application. Previous studies have concentrated on still images for the purpose of developing and evaluating models. However, in actual clinical practice, determining the current endoscopic camera position must be achieved in real time, and there have been few studies conducted for this purpose. Real-time position recognition has the potential to be a highly useful technique for determining the location of detected lesions or for developing notification functions for excessive examination. Using a prediction model trained on still images for real-time prediction can result in highly unstable outcomes, as it evaluates each frame or image independently. Nam et al. collected capsule endoscopy data to develop an image-based learning model, which was then applied to video to classify the current endoscopic position in the stomach, small intestine, or colon [19]. They employed a convolutional neural network (CNN)-based EfficientNet model along with a long short-term memory (LSTM) layer to learn the temporal dependencies within the video data. To address the bouncing values that occurred in the model results when applied to video, they performed probability calibration by applying a gaussian filter based on a gaussian distribution. Therefore, applying a separate algorithm or processing method is necessary when validating with video data.

**Figure 1:**
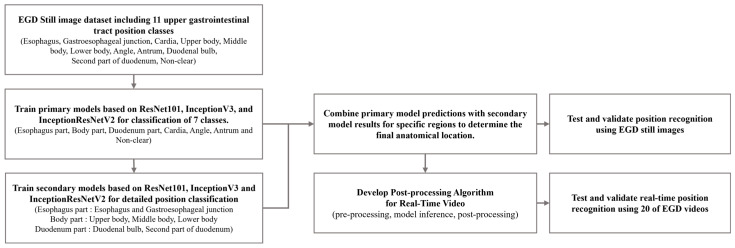
Flowchart of deep learning training with EGD still image dataset and location inference for EGD videos.

In this study, we propose the development of a fine-grained position prediction model for anatomical locations in the gastrointestinal tract using only still images. The flowchart of this study is shown in Figure 1. The model was configured to predict 11 anatomical locations: esophagus (ES), gastroesophageal junction (GE), cardia (CR), upper body (UB), middle body (MB), lower body (LB), angle (AG), antrum (AT), duodenal bulb (BB), second part of the duodenum (SD), and non-clear parts (NO). A dataset of still gastric images was then constructed. To achieve more detailed location recognition, we combined images of similar regions into single-part classes to train the primary model. Subsequently, we developed a secondary model to classify the combined classes into individual classes. By applying the results from both models, we obtained final predictions for the 11 anatomical locations. Using a test dataset composed of single images, we verified the prediction performance of each trained model and compared the final prediction results with the actual locations. This allowed us to confirm that the camera’s position during an examination can be recognized using only still images from gastroscopy.

Additionally, the primary purpose of this study was to develop a prediction model from a single image that can be used in real time. To achieve this, it was necessary to conduct a thorough evaluation of the model’s predictions from individual images. Therefore, we propose a post-processing algorithm for real-time video recognition. To test the efficacy of this proposed algorithm, we collected 20 gastroscopy video datasets and used them for testing and verification. This allowed us to assess the real-time location recognition performance of the proposed algorithm.

## 2. Materials and Methods

### 2.1. Data Acquisition

We collected 31,403 still images from a total of 1000 patients (664 males and 336 females) with no abnormal findings and who underwent EGD at Gachon University Gil Hospital between January 2018 and December 2021. Abnormal findings in patients were excluded if they included gastric cancer, gastric ulcer, or gastric adenoma. Conversely, patients with intestinal epithelialization or atrophic gastritis were considered normal. To ensure the anonymity of the patients, the data were anonymized. The patients were distributed across a range of age groups, with the following numbers in each group: 19–29 years (6), 30–49 years (239), 50–64 years (519), and over 65 years (236). A total of 20 EGD videos were collected, recording the endoscopy from the time the camera was inserted to the time the doctor finished the examination and removed the camera from the body, with an average length of 2 min and 57 s. This study was conducted in accordance with the Declaration of Helsinki and approved by the Institutional Review Board of Gachon University Gil Medical Center, which waived the requirement for informed consent from the participants (IRB NO. GBIRB2021-383).

### 2.2. Experimental Environments

This study was developed using the Python-based TensorFlow (version 2.3.0) library, and the research was conducted on an Ubuntu 18.04.6 LTS operating system with an Intel(R) Xeon(R) CPU E5-1650 v4 @ 3.60 GHz processor and a Titan Xp GPU (NVIDIA, Santa Clara, CA, USA). Furthermore, the image and video operations were conducted using the OpenCV (version 4.5.5.64) library. The EGD still images were labeled using Image J (version 1.52i) software, while the EGD videos were labeled using AnnoVie software (version 1.0.6.727, MTEG, Seoul, Republic of Korea).

### 2.3. Data Labeling

Two gastrointestinal experts with specialized medical knowledge performed the labeling of the gastrointestinal anatomical positions of the still image and video datasets. Unnecessary areas of the still images and frame images of the video data were cropped, including the patient information, acquisition equipment information, etc.

In developing our labeling classes of the gastrointestinal anatomical positions, we followed the established guidelines for endoscopic photo-documentation, which are integral to quality assurance in endoscopic procedures. As detailed by the European Society of Gastrointestinal Endoscopy in 2001 [20], upper gastrointestinal (GI) endoscopic photo-documentation emphasizes the capturing of standardized images of key anatomical landmarks. This approach ensures that significant areas are documented, thereby avoiding the omission of critical details during examinations. These guidelines emphasize the importance of capturing a minimum of eight sites in standard EGD exams to reduce variability in documentation and enhance inspection quality. Our labeling classes were chosen based on the critical anatomical landmarks emphasized in these guidelines: esophagus, gastroesophageal junction, cardia, upper body, middle body, lower body, angle, antrum, duodenal bulb, second part of the duodenum, and non-clear parts.

**Figure 2:**
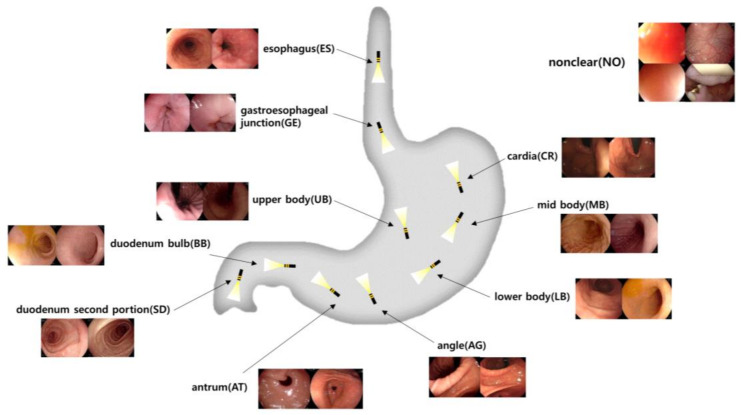
Visualization of the upper gastrointestinal anatomical positional classifications of still image data obtained from EGDs.

The esophagus is the muscular tube that transports food from the mouth to the stomach. The gastroesophageal junction marks the transition from the esophagus to the stomach. The cardia is the entry point into the stomach. The upper body refers to the upper part of the stomach, followed by the middle body and lower body, which are the central and lower parts, respectively. The angle is the angular notch of the stomach. The antrum is the lower portion that grinds food and moves it toward the small intestine. The duodenal bulb is the first part of the duodenum where the stomach contents mix with digestive enzymes, followed by the second part of the duodenum, where digestion continues in the small intestine. The non-clear class addresses situations where the image quality is insufficient to identify specific landmarks due to factors like rapid manipulator speed or the camera being too close to the stomach wall, which aligns with the guidelines’ focus on complete and clear images. Implementing these labeling classes not only facilitates standardization in image capture but also supports potential computer-aided systems that could automate photo-documentation, assisting endoscopists in achieving consistent, high-quality examinations regardless of individual variability. A visualization, including the structure and sample images of the defined anatomical landmarks in the gastrointestinal tract, is shown in Figure 2.

The esophagus and gastroesophageal junction, the upper, middle, and lower parts of the stomach, and the duodenal bulb and second portion of the duodenum are connected structures with visually similar features, blurring clear classification of the region. Therefore, we first cleaned up the primary classification criteria by lumping classes with ambiguous visual features (i.e., unclear boundaries) into one category. We then secondarily classified the dataset into subcategories, as shown in Table 1.

It was necessary to specify the ground truth of each frame to evaluate the EGD video data for a model that was trained using still images. However, the labels were assigned in seconds due to the ambiguity of the start and end of the anatomical locations. Furthermore, points of change in the anatomical positions were ambiguous, and therefore, a clear position could not be assigned. Consequently, a frame range was defined to allow for multiple positions. The frame range was set to 3 s with 180 frames, based on the video data at 60 fps.

### 2.4. Classification Model for Gastrointestinal Anatomical Positions

For the training and evaluating of the gastrointestinal anatomical position classification models, we aimed to use a ratio of 8:1:1 for the training, validation, and test datasets. In addition, to avoid class imbalance in the overall dataset, the datasets of each class were randomized according to a fixed ratio. In the case of the primary classification dataset, the data from similar regions were combined into one class, which may have caused imbalance among the classes, and the metrics of performance may not have been objective. To overcome this problem, 180 images were under-sampled for each class to create a test dataset, and the remaining images were divided according to the ratio. The datasets divided by each class were combined into training and validation datasets to build the final dataset.

To develop a model for predicting the anatomical positions of the gastrointestinal tract, several CNN models were selected for consideration. These included the ResNet101, InceptionV3, and InceptionResNetV2 models, which are commonly utilized in image classification tasks. The performance of these models was then compared. The hyperparameters were 300 epochs, a batch size of 32, the optimizer stochastic gradient descent (SGD), an initial learning rate of 1 × 10^−5^, and ReduceLROnPlateau (factor = 0.1, patience = 10) as the learning rate scheduler [21]. We also performed transfer learning using ImageNet weights supported by Keras [22]. We also resized the ResNet101 data to 224 and the InceptionV3 and InceptionResNetV2 data to 299 for use as pretrained weights.

### 2.5. Post-Processing Algorithm for Real-Time Video

In this study, the deep learning model utilized three-dimensional image data. However, the video data added the dimension of time, making them four-dimensional. For example, each video frame consists of a width, height, and color channels, and these frames are ordered sequentially over time. This setup allows video data to provide continuous changes along the time axis. To perform inference at the video level, each frame was processed independently, meaning the model made predictions on a frame-by-frame basis. However, since consecutive frames often have minimal pixel changes, adjacent frames are likely to produce very similar results. To reduce redundancy and focus on meaningful changes, we opted for a fixed frame sampling interval rather than processing every single frame.

In this study, we sampled the video at a rate of 20 frames apart in a 60 fps (frames per second) video, effectively using 3 frames per second for inference. By selecting 3 frames per second, the model captured the changes in position in real time while reducing unnecessary repetition. This allowed the model to perform inference on frames that showed more distinct changes, enabling efficient and meaningful position predictions for the video data.

The initial step involved processing each frame of the gastroscopy video with a specifically developed CNN model. In this process, the primary classification model categorized each frame into general anatomical regions. For instance, the gastric bodies (upper, middle, and lower) were grouped into a single part. Based on the primary classification results, the secondary classification model was then applied to further refine the specific locations within that part. This secondary model classified the general parts into more detailed sub-regions, such as the upper, middle, or lower bodies, providing more precise location predictions.

**Figure 3:**
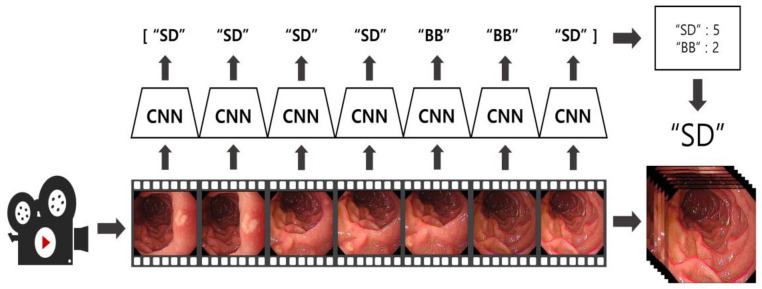
Visualization of the EGD video application algorithm using the CNN-based EGD still image classification model.

The results from each frame were stored alongside the predictions of the previous 6 frames, and the final location was determined using a hard voting method [23]. By selecting the most frequently predicted class among the 7 frames, the algorithm maintained stability and consistency, even during rapid position changes. Figure 3 illustrates this algorithm. Frames from the video data were sampled at intervals of 20 frames and fed into the model. The final location predictions were adjusted based on a hard voting process that included the current frame and the previous six predictions. Every 20 frames, the new frame’s prediction was sequentially added to the existing voting data, enabling continuous inference over time.

### 2.6. Model Performance Evaluation

To evaluate the performance of the trained location prediction model, we used an independent dataset consisting of still images not included in the training process. The test dataset for the primary classification model comprised a total of 1260 images, with 180 images randomly selected for each class to minimize any potential performance evaluation bias due to class imbalance. For the secondary classification models, the esophagus and gastroesophageal junction classification model and the duodenal bulb and duodenum second portion classification model each used a total of 176 images, with 88 images selected per class. The model for classifying the three parts of the gastric body (upper, middle, and lower) used 191 images per class, totaling 573 images for validation.

The model was applied to 20 gastroscopy video datasets to validate its performance. Data were extracted from each video from the point where the endoscope entered the mouth until the end of the examination, when the instrument exited the mouth. Using these video data, we conducted performance verification of both the model and the developed post-processing algorithm, comparing real-time prediction accuracy with and without the post-processing algorithm applied.

For evaluation with still images, we used metrics such as the accuracy, precision, recall, F1 score, and AUC. For real-time processing with video data, sensitivity and specificity were used as the performance metrics. These metrics were calculated using the mathematical formulas provided in Equations (1)–(5), with the evaluation components of true positive (TP), true negative (TN), false positive (FP), and false negative (FN) represented by a confusion matrix [24,25]. The evaluation of the EGD videos was performed frame-by-frame according to the ground truth labels assigned to each frame, with TP, TN, FP, and FN derived from the inference results. However, if the algorithm’s inference result was classified as the NO class, the frame was excluded from evaluation because the model inferred it as a visually unclear region.

(1) $$Accuracy=\frac{TP}{TP+FP+FN+TN}$$

(2) $$Precision=\frac{TP}{TP+FP}$$

(3) $$Recall\left(Sensitivity\right)=\frac{TP}{TP+FN}$$

(4) $$F1\;Score=2\times \frac{Precision\times Recall}{Precision+Recall}$$

(5) $$Specificity=\frac{TN}{TN+FP}$$

## 3. Results

### 3.1. Evaluation of Model on Still Images

A comparison of the performance of CNN models was carried out using a test dataset that was not employed during the training phase. Its purpose was to verify the performance of the position prediction learning model on still images. A comparison of the models is shown in Table 2.

**Figure 4:**
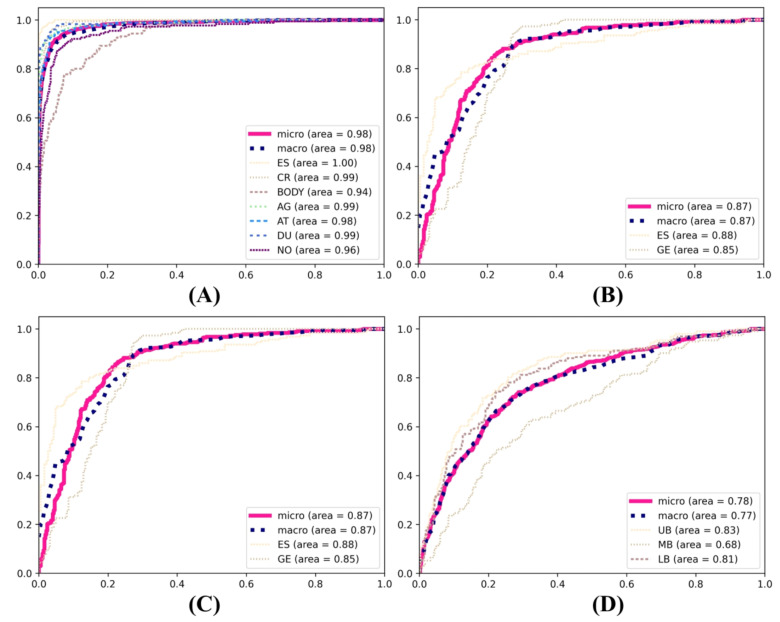
ROC curves of the primary and secondary deep learning models. (**A**) Esophagus part, cardia, gastric bodies part, angle, antrum, duodenum part, and non-clear. (**B**) Esophagus and gastroesophageal junction. (**C**) Upper body, middle body, and lower body. (**D**) Duodenal bulb and duodenum second portion.

In the primary classification tasks pertaining to the anatomical positioning of the gastrointestinal tract, the InceptionV3 model exhibited consistent superiority in the evaluation of the training models. It achieved the highest F1 score (85.24%), precision (85.33%), and recall (85.32%) in the primary classification, outperforming both InceptionResNetV2 and ResNet101. InceptionResNetV2 also demonstrated a commendable performance, with an F1 score of 84.67%, precision of 84.65%, and recall of 84.76%. In comparison, ResNet101 exhibited relatively weaker performance, with an F1 score of 75.58%, precision of 75.71%, and recall of 75.67%. In the secondary classification tasks, InceptionV3 demonstrated superior performance in the esophageal–gastroesophageal junction classification, attaining an F1 score of 83.54%, precision of 84.65%, and recall of 83.60%. In comparison, ResNet101 exhibited a comparatively lower performance, with an F1 score of 77.15%, precision of 77.21%, and recall of 77.11%. In the classification of the upper body, middle body, and lower body, InceptionResNetV2 demonstrated the highest performance, with an F1 score of 59.45%, precision of 59.50%, and recall of 60.21%. InceptionV3 also demonstrated robust performance, with an F1 score of 57.35%, precision of 57.77%, and recall of 57.77%. In the classification of the duodenal bulb and duodenum second portion, InceptionResNetV2 demonstrated the highest performance, with an F1 score of 93.17%, precision of 93.22%, and recall of 93.18%. The ROC curve of the still image models is shown in Figure 4.

By applying the test data to the primary position classification model, we obtained the secondary position classification results for the images predicted by the secondary position classification model in the corresponding classes. This process allowed us to obtain the position results for the final dataset of all 11 anatomical position classes. In assessing the performance metrics of various convolutional neural network architectures, as shown in Table 3, InceptionV3 showed superior efficacy in comparison to ResNet101 and InceptionResNetv2. InceptionV3 achieved an F1 score of 79.79%, precision of 80.57%, and recall of 80.08%.

In addition, the sensitivity and specificity of the results for each class when applying the InceptionV3 model, which showed the highest performance in the above experiment, are shown in Table 4. The SD class showed the highest performance with sensitivity of 92.44% and specificity of 98.42%, and the AG class showed sensitivity of 90.62%. The UB class showed the lowest performance with sensitivity of 37.88% and specificity of 98.57%.

### 3.2. Evaluation of a Gastrointestinal Anatomical Position Prediction Model with Endoscopy Video Data

We selected the appropriate number of consecutive frames to perform hard voting and apply the post-processing algorithm. The post-processing algorithm, which predicts the final class information using information from consecutive frames, was evaluated using 4, 7, 10, and 13 frames. The seven-frame configuration, which demonstrated the highest performance, as shown in Table 5, was chosen as the basis for hard voting.

For frame-by-frame anatomical position prediction in the gastroscopy videos, each frame extracted from the video was applied to the training model to obtain its position. The prediction results of the model were compared, as shown in Table 6, and evaluated by comparing the position prediction results obtained from all frames with the correct labeling answers for each point in time. For the ResNet101 model, the F1 score was 45.15% to 47.87%, the precision was 61.46% to 61.74%, and the recall was 45.76% to 47.79%, depending on the post-processing applied. For the InceptionV3 model, the F1 score was 55.12% to 59.66, the precision was 69.63% to 70.07%, the recall was 54.69% to 59.07%. For the InceptionResNetV2 model, the F1 score was 56.25% to 61.37%, the precision was 69.51% to 73.08%, and the recall was 54.23% to 57.21%.

The sensitivity and specificity of the best-performing InceptionResNetV2 model applied to the EGD videos in the above experiment are shown in Table 7 for each class. Based on the all-video dataset, the esophagus showed the highest results, with sensitivity of 91.64% and specificity of 96.05%, while the gastroesophageal junction showed the lowest results, with sensitivity of 55.45% and specificity of 98.84%.

## 4. Discussion

In this study, we aimed to address issues such as failure to detect lesions during EGD due to blind spots and failure to diagnose cancer. We constructed a dataset of classified EGD still images based on anatomical location features and trained ResNet101, InceptionV3, and InceptionResNetV2 models to improve their clinical utility in diagnosing lesions.

We emphasized the significance of our dataset comprising 11 anatomical location classes, including the non-clear class, which included images with resolution degradation or position ambiguity. This classification enabled the utilization of clearer and more obvious anatomical features for model training, particularly emphasizing the importance of the non-clear class in EGD video applications. Inferencing and excluding ambiguous regions outside of the detailed observation points in EGD videos contributed to enhancing the position reading reliability.

The performance evaluation of InceptionV3, InceptionResNetV2, and ResNet101 on still images revealed InceptionV3 as the best-performing model, with an F1 score of 79.79%, precision of 80.57%, and recall of 80.08%. However, it struggled to learn to classify the gastric body region, which is a visually ambiguous region, and this affected its performance in certain classes, as seen in Table 3. This can be attributed to the high visual similarity among the upper, middle, and lower body classes. Similar patterns were observed in the EGD video application, emphasizing the necessity for a sophisticated deep learning model and a more refined dataset, as seen in Table 6. When processing video, extracting images from all frames and applying them to a model is likely the most accurate way to showcase the model’s performance. However, using all frames on a model trained on single images results in independent predictions for each frame, leading to unstable position recognition. In this study, we selected a frame sampling size of 20 frames to apply to the model, allowing it to detect sufficient position changes from a continuously moving endoscopic device and verify the model’s real-time position prediction results. Additionally, post-processing generally improved the performance by more than 4%, highlighting the algorithm’s effectiveness in evaluating all frames and reducing errors occurring in a small subset of frames.

Improvements are needed for the post-processing algorithm to facilitate real-time applications using video data. The current algorithm, which utilizes hard voting, may yield entirely incorrect predictions if the performance of the prediction model declines. This is especially problematic when the endoscopic view becomes obscured by bubbles or water during examination, as most frames should ideally be classified as non-clear in such cases. However, the algorithm tends to retain the previous class’s result, which degrades the overall accuracy. To address this, as seen in prior research, we plan to enhance the system by incorporating primary post-processing techniques for frame predictions or by developing algorithms or models that determine whether to apply the location prediction model based on image quality. These improvements aim to ensure stable gastrointestinal location predictions even under challenging conditions. Despite these efforts, the most crucial aspect of video data prediction remains the temporal dependency or time-series characteristic. Unlike single-image prediction models that treat each input independently, time-series data can achieve higher accuracy by considering the flow of time. To address this, we aim to enhance performance by utilizing large-scale video data and developing a new gastrointestinal location prediction model. This model will incorporate architectures that reflect temporal dependencies, such as a 3D CNN or a combination with LSTM, allowing for more accurate and reliable predictions.

Despite collecting EGD data from 1000 patients to enhance model generality, limitations exist in potentially collecting similar data patterns due to restricted endoscopy machines and operators. Our future studies will aim to secure generalizability through multi-institutional data collection with varied endoscopy devices and operators. We also intend to introduce more detailed criteria for anatomical locations, enhancing classification performance. This refinement of dataset criteria is expected to decrease the non-clear patterns, thereby improving the reliability and generalizability of EGD video application. Furthermore, in this study, the model was trained on 1000 normal patients, potentially biasing the results toward clear data with few distractions. For example, the model may struggle to make accurate predictions in various clinical environments or tasks, such as when actual lesions are present, when images are converted to narrow-band imaging for diagnosis, when tissue is stained, or when instruments are used for biopsies. In future endeavors, we will construct additional datasets containing lesions for incremental training, aiming to improve the model’s sensitivity to detecting abnormalities.

## 5. Conclusions

The purpose of this study was to enhance the precision and dependability of endoscopic examinations by developing a deep learning-based model that can classify anatomical locations in real time during endoscopic procedures. The findings of this study demonstrate that the learning process from EGD still images classified according to a sophisticated anatomical classification is applicable to location inference in EGD videos, even when many distracting features are present. However, this study also underscores the necessity for comprehensive investigation into specific challenging categories to enhance the precision of EGD image location inference and address the challenge of cancer diagnosis posed by blind spots in clinical EGD examinations. By optimizing training data and models, we seek to contribute to the advancement of quality healthcare by reducing the resources required for EGD screening and improving diagnostic outcomes. This technological advancement has the potential to reduce physician fatigue, support medical staff in diagnosis, and ultimately provide better healthcare services to patients. Combining lesion detection technology, which is being actively researched in the field of gastroscopy, with the ability to recognize the location of these lesions, can be crucial for developing various advanced medical technologies. This includes automatic report generation, follow-up examinations for patients requiring continuous monitoring, and other innovative healthcare applications. By focusing on accurate and dependable anatomical localization, this research aims to support clinicians in making precise diagnoses and improve overall endoscopic examination quality, ultimately contributing to better patient outcomes.

## Figures and Tables

**Table 1:** Distribution of upper gastrointestinal anatomical positional classifications of still image datasets obtained from EGDs.

Data Categorization Criteria	Number of Image Data (%)	
	Primary Classification	Secondary Classification
Esophagus	6910 (22.0)	4707 (68.1)
Gastroesophageal junction		2203 (31.8)
Cardia	2126 (6.7)	
Upper body	8815 (28.0)	3046 (34.5)
Middle body		3521 (39.9)
Lower body		2248 (25.5)
Angle	2226 (7.0)	
Antrum	4161 (13.2)	
Duodenal bulb	2631 (8.3)	1036 (39.3)
Duodenum second portion		1595 (60.6)
Non-clear	4534 (14.4)	
Total	31,403 (100.0)	

**Table 2:** Results of primary and secondary classifications of various models using the EGD still image dataset.

		Accuracy (95% CI)	F1 Score (95% CI)	Precision (95% CI)	Recall (95% CI)	AUC (95% CI)
Primary classification	Esophagus Part, Cardia, Gastric Bodies Part, Angle, Antrum, Duodenum Part, and Non-Clear					
	ResNet101	75.68 (73.33–78.02)	75.58 (73.20–77.92)	75.71 (73.35–78.06)	75.67 (73.33–78.02)	95.26 (94.50–95.95)
	InceptionV3	85.35 (83.41–87.22)	85.24 (83.26–87.17)	85.33 (83.32–87.28)	85.32 (83.41–87.22)	97.63 (97.13–98.10)
	InceptionResNetV2	84.75 (82.70–86.75)	84.67 (82.53–86.62)	84.65 (82.48–86.66)	84.76 (82.70–86.75)	97.82 (97.32–98.29)
Secondary classification	Esophagus and Gastroesophageal Junction					
	ResNet101	77.11 (72.85–81.45)	77.15 (72.83–81.45)	77.21 (72.85–81.45)	77.11 (72.85–81.45)	82.70 (87.48–86.45)
	InceptionV3	83.65 (79.83–87.37)	83.54 (79.52–87.34)	84.65 (81.13–88.23)	83.60 (79.83–87.37)	88.59 (85.10–91.75)
	InceptionResNetV2	82.01 (77.95–85.75)	81.94 (77.79–85.72)	82.64 (78.60–86.14)	81.99 (77.95–85.75)	86.60 (82.82–90.04)
	Upper Body, Middle Body, and Lower Body					
	ResNet101	51.58 (47.47–55.67)	50.72 (46.62–54.97)	51.12 (46.82–55.41)	51.48 (47.47–55.67)	70.25 (67.02–73.33)
	InceptionV3	57.71 (53.75–61.95)	57.35 (53.35–61.66)	57.77 (53.36–61.71)	57.77 (53.75–61.95)	74.68 (53.75–61.95)
	InceptionResNetV2	60.16 (56.02–64.22)	59.45 (55.15–63.49)	59.50 (55.21–63.58)	60.21 (56.02–64.22)	77.31 (74.25–80.11)
	Duodenal Bulb and Duodenum Second Portion					
	ResNet101	83.60 (78.39–88.64)	83.52 (78.26–88.65)	83.79 (78.43–89.03)	83.52 (78.39–88.64)	92.96 (89.11–96.21)
	InceptionV3	89.81 (85.23–94.32)	89.78 (85.22–94.32)	89.91 (85.23–94.34)	89.77 (85.23–94.32)	96.85 (94.56–98.73)
	InceptionResNetV2	93.07 (89.20–96.59)	93.17 (89.18–96.59)	93.22 (89.23–96.62)	93.18 (89.20–96.59)	97.21 (94.95–99.02)

**Table 3:** Results of the training model’s predictions of gastrointestinal anatomical positions for all 11 classes with primary and secondary classification models.

	Accuracy (95% CI)	F1 Score (95% CI)	Precision (95% CI)	Recall (95% CI)
ResNet101	68.40 (66.00–70.90)	67.29 (64.72–70.04)	67.29 (64.52–70.05)	68.41 (65.87–70.87)
InceptionV3	80.10 (77.90–82.10)	79.79 (77.36–82.09)	80.57 (78.20–82.92)	80.08 (77.70–82.30)
InceptionResNetV2	78.00 (75.70–80.30)	77.72 (75.31–80.09)	78.28 (75.74–80.68)	78.02 (75.71–80.24)

**Table 4:** Class-wise results of applying InceptionV3 model to classify still image dataset.

Data Categorization Criteria	Sensitivity (95% CI)	Specificity (95% CI)	Data Categorization Criteria	Sensitivity (95% CI)	Specificity (95% CI)
Esophagus	79.49 (72.46–86.57)	99.91 (99.73–100.00)	Angle	90.62 (86.23–94.59)	97.97 (97.10–98.71)
Gastroesophageal junction	87.23 (77.19–95.24)	97.60 (96.76–98.40)	Antrum	88.30 (83.62–92.68)	88.30 (96.61–98.42)
Cardia	90.00 (85.56–94.09)	97.07 (96.02–98.02)	Duodenal bulb	68.18 (56.34–80.00)	98.42 (97.67–99.08)
Upper body	37.88 (27.14–49.33)	98.57 (97.81–99.24)	Duodenum second portion	92.44 (87.13–96.77)	98.42 (98.87–99.82)
Middle body	43.84 (31.14–56.90)	98.50 (97.75–99.09)	Non-clear	79.81 (74.21–85.96)	95.33 (94.13–96.56)
Lower body	55.88 (40.62–71.05)	97.54 (96.63–98.37)			

**Table 5:** Results of applying different ranges of consecutive frames.

	F1 Score (95% CI)	Precision (95% CI)	Recall (95% CI)
4 Frames	59.13 (41.63–75.24)	75.60 (53.07–90.10)	57.81 (40.34–75.00)
7 Frames	61.37 (41.08–76.86)	73.08 (51.70–87.79)	57.21 (39.33–77.18)
10 Frames	59.84 (38.42–76.31)	72.15 (49.84–85.00)	57.15 (37.14–77.01)
13 Frames	55.69 (36.93–74.97)	65.97 (47.26–82.28)	56.72 (35.53–75.52)

**Table 6:** Results of applying the EGD video using various EGD still image models.

	Post-Processing	F1 Score (95% CI)	Precision (95% CI)	Recall (95% CI)
ResNet101	Without post-processing	45.15 (26.36–55.05)	61.46 (44.20–73.23)	45.76 (27.49–54.05)
	With post-processing	47.87 (21.03–61.03)	61.74 (39.62–82.06)	47.79 (24.92–59.21)
InceptionV3	Without post-processing	55.12 (38.73–71.83)	69.63 (46.56–86.52)	54.69 (39.29–66.58)
	With post-processing	59.66 (40.76–76.82)	70.07 (43.35–91.28)	59.07 (42.10–74.32)
InceptionResNetV2	Without post-processing	56.25 (39.25–73.20)	69.51 (49.78–86.92)	54.23 (38.34–72.08)
	With post-processing	61.37 (41.08–76.86)	73.08 (51.70–87.79)	57.21 (39.33–77.18)

**Table 7:** Evaluation results on a per-class basis applied to EGD videos using InceptionResNetV2-based models trained on EGD still images.

	Top 1			Top 5			Total (*n* = 20)		
Labels	Sensitivity	Specificity	Avg. Frames	Sensitivity	Specificity	Avg. Frames	Sensitivity	Specificity	Avg. Frames
ES	100.00	98.10	280	91.09	95.07	462	89.79	95.68	650
GE	65.00	100.00	400	54.96	98.14	318	52.11	98.64	345
CR	81.13	99.54	1060	78.43	96.46	564	57.41	98.05	600
UB	32.00	99.35	500	26.40	95.91	655	19.27	95.88	248
MB	38.88	95.80	720	19.15	97.97	440	19.36	95.88	1218
LB	66.66	96.53	540	25.39	98.08	420	27.22	93.37	811
AG	96.80	93.41	1880	69.95	95.76	573	65.18	95.07	525
AT	76.35	93.84	2960	77.72	96.13	1188	74.10	93.45	955
BB	78.94	95.74	380	75.78	95.96	192	56.69	95.27	216
SD	83.01	100.00	1060	74.03	99.44	426	78.55	99.18	292

## Data Availability

The data are not publicly available due to privacy or ethical restrictions.
